# Sepsis Induces Physical and Mental Impairments in a Mouse Model of Post-Intensive Care Syndrome

**DOI:** 10.3390/jcm10081593

**Published:** 2021-04-09

**Authors:** Yoshihisa Fujinami, Shigeaki Inoue, Yuko Ono, Yusuke Miyazaki, Kazumichi Fujioka, Kimihiro Yamashita, Joji Kotani

**Affiliations:** 1 Department of Disaster and Emergency and Critical Care Medicine, Kobe University Graduate School of Medicine, Kobe 650-0017, Japan; greatyoppie@yahoo.co.jp (Y.F.); windmill@people.kobe-u.ac.jp (Y.O.); m_family0222@yahoo.co.jp (Y.M.); kotanijo0412@gmail.com (J.K.); 2 Department of Surgery, Division of Gastrointestinal Surgery, Kobe University Graduate School of Medicine, Kobe 650-0017, Japan; fujiokakazumichi@hotmail.co.jp; 3 Department of Pediatrics, Kobe University Graduate School of Medicine, Kobe 650-0017, Japan; kiyama@med.kobe-u.ac.jp

**Keywords:** post-intensive care syndrome, physical impairments, cognitive impairment, mental impairment, sepsis, survivor

## Abstract

Post-intensive care syndrome (PICS) is a physical, cognitive, and mental impairment observed in intensive care unit (ICU) survivors. Although this is an emerging problem in the ICU, how sepsis induces the characteristic symptoms of PICS remains unclear. To develop a model of PICS, we induced sepsis in male C57/B6 mice via sublethal cecum slurry injection and subsequently treated them using ICU-like interventions. At 1–2 weeks post-sepsis induction, we simultaneously evaluated the abilities of the surviving mice using the following behavioral tests: (1) a grip strength test (GST) and a treadmill test for physical assessment, (2) a novel object recognition test (NORT) for cognitive assessment, and (3) an open field test (OFT) and a marble burying test (MBT) for mental assessment. The surviving mice showed a range of deficits, including muscle weakness with significantly decreased grip strength in the GST; decreased total mileage during the treadmill test; anxiety and decreased activity, with significantly decreased time in the central area, and increased duration of immobility in the OFT; and an increased number of buried marbles in the MBT. Given these physical and mental impairments in the surviving mice, our model has the potential to elucidate mechanistic insights and to discover therapeutic targets and new interventions for PICS.

## 1. Introduction

The long-term outcomes of critical illnesses are becoming an emerging problem. The expanding aging population requires more critical care support to reduce mortality [1,2,3]. Post-intensive care syndrome (PICS) involves physical, cognitive, and mental impairments that occur during intensive care unit (ICU) stay or after ICU/hospital discharge and impairs the long-term prognosis of these patients [4].

Sepsis is a severe, life-threatening acute organ dysfunction that occurs after infection [5]. Approximately 50% of patients with sepsis recover, one-third die within a year, and one-sixth present severe, persistent impairments [6]. Common post-sepsis complications include muscle weakness, fatigue, difficulty swallowing, cloudy thinking, difficulty concentrating, poor memory, difficulty sleeping, depression, and anxiety [6,7]. The prevalence of moderate-to-severe cognitive impairment is 10.6% higher among patients with severe sepsis compared to those without sepsis [8]. These findings demonstrate the critical need for improving long-term post-sepsis outcomes.

Despite the increasing focus on long-term outcomes in sepsis survivors in the field of emergency and intensive care medicine, it remains unclear whether and how sepsis induces the physical, cognitive, and mental impairments observed in PICS. This study aimed to simultaneously evaluate physical, cognitive, and mental disorders in a mouse model of sepsis.

## 2. Materials and Methods

All animal experiments were approved by the Committee on the Ethics of Animal Experiments of Kobe University Graduate School of Medicine (permit number: P180806/P190801). All experiments were conducted according to the recommendations of the International Expert Consensus Initiative for the Improvement of Animal Modeling in Sepsis [9].

### 2.1. Preparation of Cecal Slurry

Mice were intraperitoneally (i.p.) injected with cecal slurry (CS), which was prepared as previously described [10], to induce polymicrobial sepsis [11,12]. Institute of Cancer Research male mice (8–12 weeks old) were sacrificed and whole ceca were harvested. The mouse cecum was nicked and ground using a 70-µm nylon mesh cell strainer (Falcon, Bedford, MA, USA). Subsequently, 1–2 mL of sterile phosphate-buffered saline (PBS) was added, and the mixture was filtered twice. The mixture was collected and centrifuged at 11,000 rpm for 1 min and the supernatant was discarded. The residue was then suspended in filtered 15% glycerol-PBS to achieve a final concentration of 500 mg/mL. The CS (400–500 µL) was transferred to cryogenic biobanking tubes (Greiner Bio-one, Kremsmüster, Austria) and stored at −80 °C until subsequent use.

### 2.2. Animal Housing and Study Design

**Figure 1:**
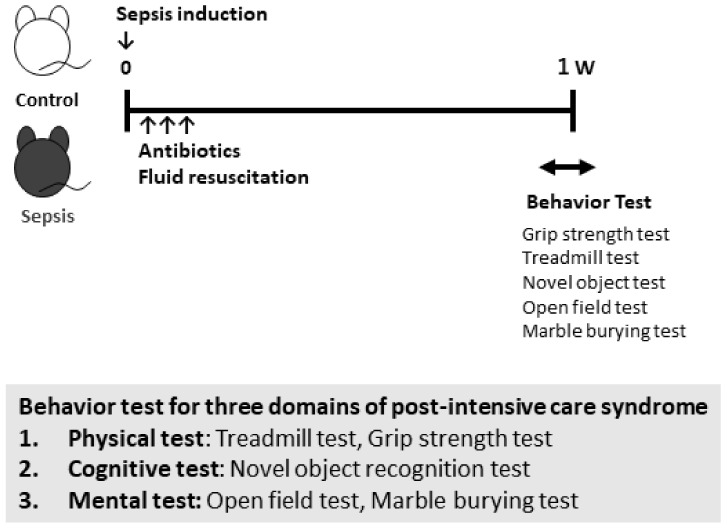
Study schema. Sepsis was induced in mice by intraperitoneal administration of 50 µL of 0.5 mg/mL cecal slurry (CS). Then, 7 to 14 days after the induction of sepsis, three animal behavioral tests were performed.

All animal experiments were conducted at the Department of Laboratory Animal Science at Kobe University. Male C57BL6J mice were obtained from CLEA Japan, Inc. (Tokyo, Japan) and housed in groups of 3–5 per cage under specific pathogen-free conditions with a 12-h light/dark cycle. During the experiments, the mice had access to food and water ad libitum. The sample sizes are shown in the figure legends. To investigate and observe whether CS injection could induce anxiety-like behavior in mice, the 10–12-week-old C57BL6J mice were randomly divided into control (injected with 15% glycerol-PBS) and CS (injected with 50 µL of 0.5 mg/mL CS) groups. Antibiotics (imipenem (IPM) 1.5 mg/mouse i.p.) and fluid resuscitation (PBS 0.9%, 30 mL/kg, subcutaneous) were administered at 24-h intervals for three days after CS injection (Figure 1).

### 2.3. Behavioral Procedures

To investigate PICS-like behaviors in the septic mice, we performed three different types of behavioral tests: (1) physical tests (grip strength test (GST) and treadmill test), (2) cognitive tests (novel object recognition test (NORT)), and (3) mental tests (open field test (OFT) and marble burying test (MBT)) (Figure 1). All behavioral tests were performed 1 week after the development of sepsis.

### 2.4. Physical Examination

#### 2.4.1. Grip Strength Test (GST)

Forelimb grip strength was measured using a grip strength meter (MK-380Si, Muromachi Kikai, Tokyo, Japan), with minor modifications to a previously described protocol [13,14]. In short, the mice used their front paws to grab a horizontal bar attached to the gauge, and their tail was slowly pulled back. Peak tension was automatically recorded at the time at which the mouse released the bar. Measurements were replicated in triplicate, and the maximum force of the three measurements was recorded. Lower grip strength was indicative of weaker muscles.

#### 2.4.2. Treadmill Test

The treadmill test was performed on a 20°-inclined mouse treadmill (category conveyor, Misumi Corp., Tokyo, Japan). We performed a modified procedure described by Seldeen et al. [15]. The animals were constantly monitored; moreover, the training speed of the exercise group was gradually increased within 2 min—specifically, from minimum (10 m/min, 1 min) to moderate (12.5 m/min, 1 min) and maximum exercise (15 m/min, 1 min). The experiment was considered complete when the mice stopped running. Finally, we calculated the accumulated distance for each speed as the total mileage. A lower total mileage was indicative of weaker muscles.

### 2.5. Cognitive Examination

#### Novel Object Recognition Test (NORT)

The NORT was conducted with some modifications to the previously described protocol [16] using an open field comprised of a clear Plexiglas box (45 cm × 45 cm × 30 cm). First, the mice were allowed to explore and habituate to the empty open field box. Next, the mice were presented with two objects, designated as A and B, for 10 min. After 30 min, the mice were exposed to two different objects, A and C, with C indicating a novel object, for 5 min. The test and training sessions were videotaped and analyzed by an experimenter who was blind to the treatment group. We recorded the length of time that the mice spent touching, sniffing, and orienting to the objects. TA, TB, and TC refer to the time spent exploring objects A, B, and C, respectively; TB/(TA + TB) and TC/(TA + TC) were calculated as recognition indexes. A lower recognition index for a new object was indicative of poor short-term memory retention.

### 2.6. Mental Examination

#### 2.6.1. Open Field Test (OFT)

The OFT is among the most frequently used animal behavior tests and is used to assess anxiety in rodents, including septic mice [17]. The open field had a size of 60 cm × 60 cm × 25 cm and was divided into nine equal squares by using lines. During the training session, the mice were placed in the left corner of the apparatus and allowed to explore for 10 min. After 30 min, the animals underwent a test session in the same open field. A video camera was placed approximately 250 cm above the center of the field and the sessions were recorded. The following parameters were analyzed: locomotion (line crossing), center square entries, and immobility. All test sessions were performed for 5 min. Lower locomotor activity and center square entry and higher immobility were indicative of greater anxiety levels.

#### 2.6.2. Marble Burying Test (MBT)

The MBT is often used to assess mouse anxiety, depression, and post-traumatic stress disorder [17]. For acclimatization, the mice were individually placed in plastic cages (25 cm × 15 cm × 17 cm) for 30 min. Then, 12 glass marbles were placed atop bedding material that was approximately 5 cm thick. After 30 min, the mice were removed, and the number of marbles covered by more than two-thirds of the bedding material was counted. A higher number of buried marbles was indicative of greater anxiety levels.

### 2.7. Statistical Analyses

All statistical analyses were performed using Easy R statistical software [18]. The normality of the data was determined using the Kolmogorov–Smirnov test. Unpaired *t*-tests were used for between-group comparisons. The log-rank test was used for survival studies. Statistical significance was set at *p* < 0.05. The results are presented as the mean ± standard error of the mean (SEM).

## 3. Results

### 3.1. Cecal Slurry Injection Induces Sublethal Outcomes in Mice

**Figure 2:**
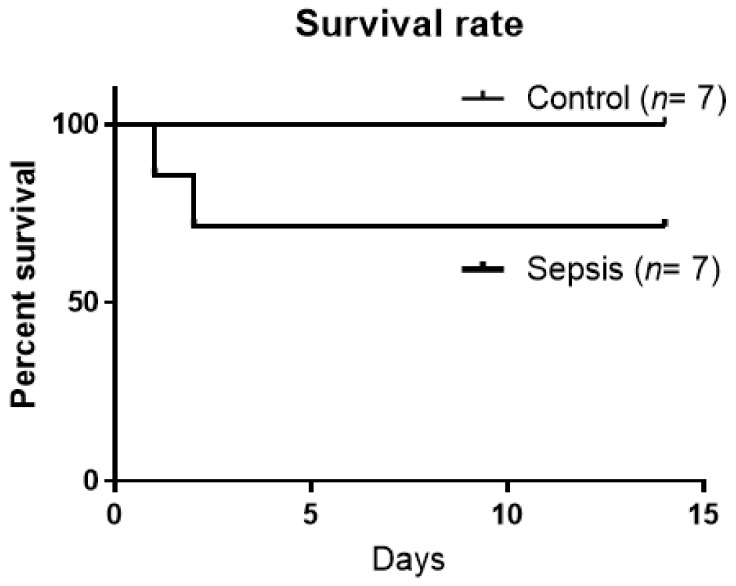
Survival after CS injection. Sepsis was induced in mice by intraperitoneal administration of 50 µL of 0.5 mg/mL cecal slurry (CS). Survival study after CS injection (*n* = 7 per group).

First, we conducted a survival study to determine whether the CS-injected mice survived sepsis. After sublethal sepsis induction by injecting 50 µL of 0.5 mg/mL cecal slurry into the abdomen, the survival rate was 73% at 14 days post-sepsis. There were no significant differences between the groups of survival (*p* = 0.07) (Figure 2).

### 3.2. Sepsis Induces Physical Impairments

**Figure 3:**
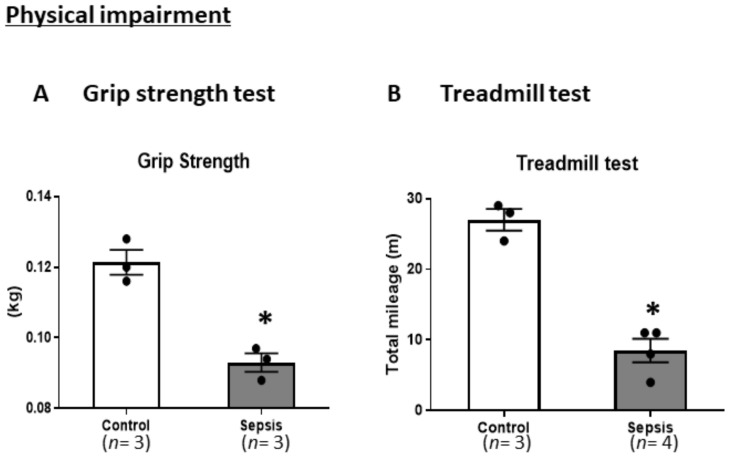
CS-induced septic mice exhibited physical impairments including muscle weakness. (**A**) Grip strength test. (**B**) Treadmill test. Data are expressed as the mean ± standard error of the mean (SEM) (*n* = 3 per group). * *p* < 0.05, vs. control.

To determine whether the mice who survived after sepsis demonstrated physical impairments, including muscle weakness, we conducted a GST and treadmill test on a different set of mice than those we used in the survival study. In the GST, we found a significant decrease in inverted grip endurance in the septic mice compared to the control mice at 7 days post-sepsis (*p* < 0.05) (Figure 3A). In the treadmill test, there was a significant decrease in total mileage at 7 and 14 days post-sepsis (*p* < 0.05). These results suggest that septic mice present with physical impairments due to muscle weakness (Figure 3B).

### 3.3. Sepsis Does Not Induce Cognitive Impairments

**Figure 4:**
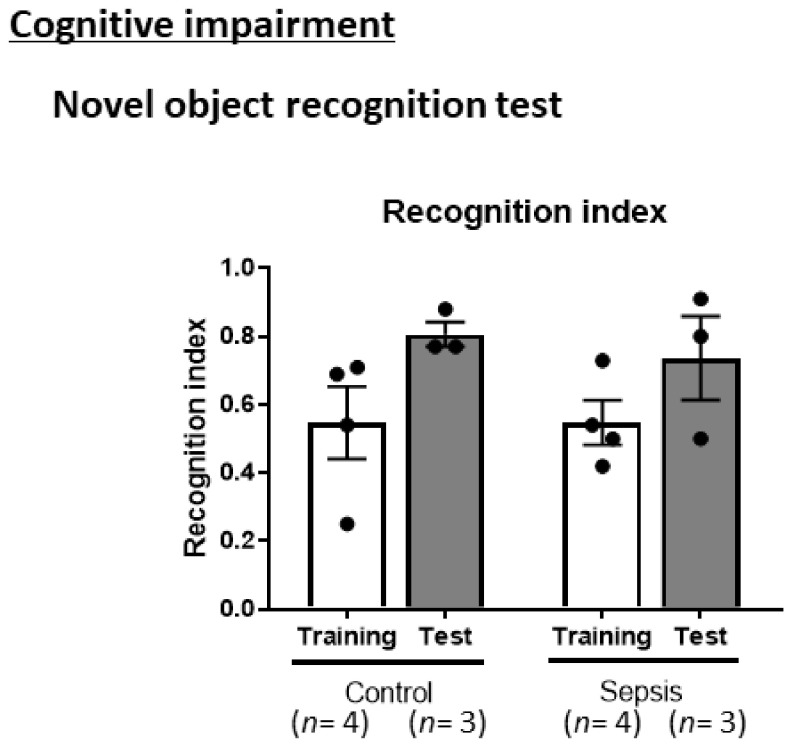
CS-induced septic mice did not exhibit short-memory impairments. Novel object recognition test. Data are expressed as the mean ± SEM (*n* = 3–4 per group).

Next, we conducted an NORT to identify cognitive impairments in the surviving mice. In the NORT, there were no differences in the recognition index between the training and test phases in the control mice (Figure 4). These results suggest that short-term memory impairments may not occur following sepsis.

### 3.4. Sepsis Induces Mental Impairments

**Figure 5:**
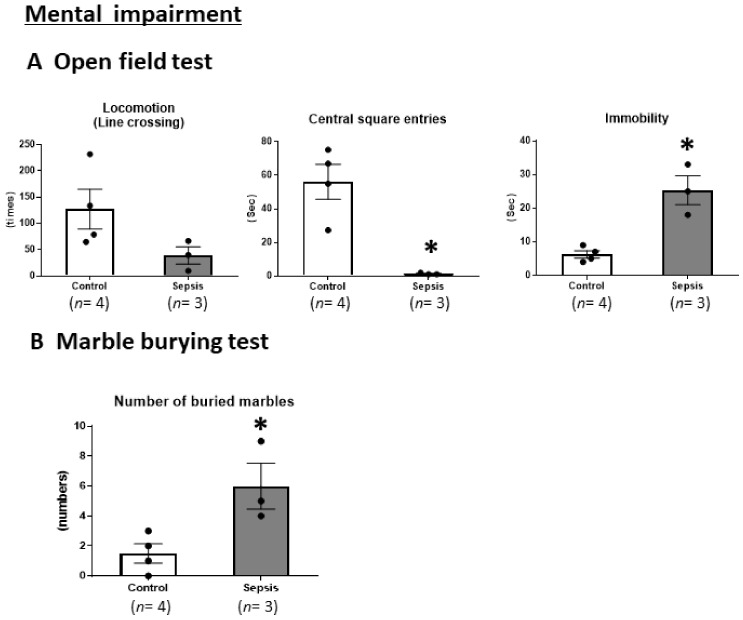
CS-induced septic mice exhibited anxiety-like behaviors. (**A**) Open field test. (**B**) Marble burying test. Data are expressed as the mean ± SEM (*n* = 3–4 per group). * *p* < 0.05, vs. control.

Finally, we performed an OFT and MBT to determine whether sepsis induced mental impairments. In the OFT, compared with the control group, the sepsis group showed a significant reduction in center square entries as well as a significant increase in the duration of immobility (*p* < 0.05). In the MBT, the sepsis mice showed a significantly higher number of buried marbles (*p* < 0.05) (Figure 5). These results suggest that sepsis induces anxiety.

## 4. Discussion

We observed muscle weakness, anxiety, and decreased activity in septic mice, which are characteristic of the symptoms observed in patients with PICS. Previous mouse studies have reported that sepsis causes skeletal muscle lactate release, skeletal muscle wasting [13], glycolysis, atrophy [19,20], and muscle proteolysis [21]. Polymicrobial peritonitis produced by cecal ligation and puncture (CLP) decreases skeletal muscle protein synthesis, partly by impairing the mammalian target of rapamycin activity [22]. However, the previous studies had short observation durations of 16 h [21], 24 h [19,22], and 7 days [19]. We found a significant decrease in the grip strength of the septic mice compared to the control mice at 7 days post-sepsis (*p* < 0.05). In the treadmill test, the septic mice also showed a significant decrease in total mileage at 7 days post-sepsis (*p* < 0.05), which is suggestive of physical impairments resulting from muscle weakness.

In this study, the septic mice showed a significant decrease in locomotion and time in the central area; moreover, they showed a significant increase in the duration of immobility in the OFT. In the MBT, the septic mice showed a significantly higher number of buried marbles, which suggests that sepsis induces anxiety. Regarding mental impairments, sepsis-associated encephalopathy (SAE) is a representative syndrome in mice. It is a diffuse central nervous system dysfunction during sepsis. Given that SAE affects > 70% of patients with sepsis and is associated with increased mortality and poor outcomes, early diagnosis and suitable interventions are vital for ameliorating the mortality and morbidity observed in patients with sepsis [23]. In a previous SAE mouse model, disruptions in energy metabolism induced behavioral and cognitive consequences of acute systemic inflammation [24], which caused cognitive impairments accompanied by selective phenotype loss of parvalbumin interneurons, as well as interleukin-1β, interleukin-18, and interleukin-6 expression [25,26]. We previously reported that infiltrated regulatory T cells and helper type-2 cells contribute to SAE attenuation and alleviate SAE-induced mental disorders by resolving neuroinflammation in the chronic sepsis phase [17].

Few previous studies have established animal models of PICS and ICU-acquired weakness (ICU-AW). Witteveen et al. assessed an ICU-AW model using in vivo strength measurements and myosin/actin assays in an *Escherichia coli* septic peritonitis mouse model [27]. Illendula et al. reported that surgery, anesthesia, and an intensive care environment impaired mouse behaviors involving attention, memory, and thought organization, which allowed for the establishment of a clinically relevant mouse model of perioperative delirium [28]. These models revealed human-like PICS symptoms; however, neither mouse model was intubated or ventilated. The most commonly used model for mimicking the ICU uses septic mice that have undergone CLP [9]. Based on a previous study using a septic model [10], we used CS-injected mice with standard sepsis treatment, including antibiotic administration and fluid resuscitation. This model can easily control the severity and allows for the establishment of a model with minor sepsis [10].

This study observed PICS-like symptoms in mice within 1 week. Although this observation period appears short, the aging rate of mice is 30 times that of humans [29]. Based on this formula, one week for mice can be calculated as 30 weeks for humans. Since patients after ICU discharge are followed up for 3–6 months to diagnose PICS symptoms, the observation period in our study was suitable for evaluating PICS-related impairments.

A recent prospective, multicenter, observational cohort study showed that physical, mental, and cognitive impairments occurred in 32.3%, 14.6%, and 37.5% of ICU survivors, respectively, after 6 months [30]. Furthermore, 63.5% of ICU survivors had one domain of PICS impairment and 17.8% had two or more domain impairments, suggesting that PICS comprises not only a single domain but also complex domains in ICU survivors [30]. Our findings suggest a significant overlap among the three broad categories of symptoms, since an individual with PICS may have symptoms in more than one category.

**Figure 6:**
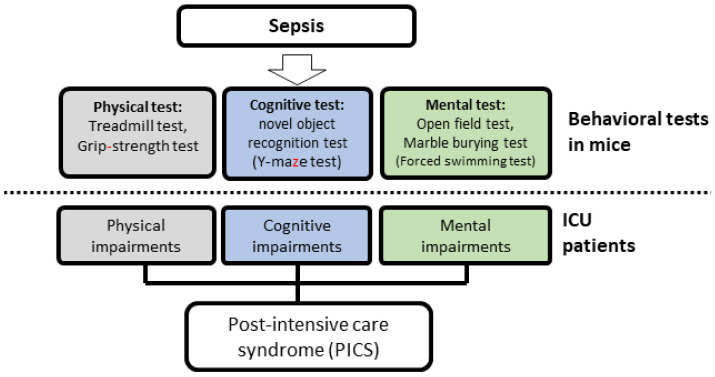
Proposal of post-intensive care syndrome (PICS) modeling using septic mice. Grip strength tests and treadmill tests mimic physical impairments in patients. The novel object recognition test and Y-maze test mimic cognitive impairments in patients. The open field test, marble burying test, and forced swimming tests mimic mental impairments in patients.

This study has several limitations. First, we performed limited behavioral testing, which included five behavioral tests (GST, treadmill test, NORT, OFT, and MBT). Second, we did not observe short-term memory impairments in the mice, although several studies have demonstrated that sepsis induces cognitive impairments [16,25]. One explanation for this is that we performed the NORT after a very short training phase (10 min), which may have been inadequate. A second explanation is that we did not conduct a priori sample size calculation, which is generally recommended for animal experiments. We speculate that the small sample size with low statistical power was the reason for the lack of significant differences between the training phase and the test phase in the control mice, which should have been significant. Third, we only performed the NORT to assess cognitive impairment. Future studies should include additional investigations, including the Y-maze test for assessing cognitive impairments as well as forced swimming tests to assess mental impairments (Figure 6).

## 5. Conclusions

Following sepsis induction, the surviving mice presented with physical and mental impairments. Long-term evaluation of muscle strength and mental health in mice surviving sepsis may help in establishing a mouse model of PICS.

## Data Availability

All relevant data are contained within this manuscript.
